# Nutritional Modulation of the Immune Response Mediated by Nucleotides in Canine Leishmaniosis

**DOI:** 10.3390/microorganisms9122601

**Published:** 2021-12-16

**Authors:** Sergi Segarra

**Affiliations:** R&D Bioiberica S.A.U., 08950 Esplugues de Llobregat, Spain; ssegarra@bioiberica.com; Tel.: +34-934-904-908

**Keywords:** leishmaniasis, canine leishmaniosis, immune response, nucleotides, AHCC, bioactive compounds, Th1 immune response, immunonutrition, One Health, zoonoses

## Abstract

Leishmaniasis is an emerging, uncontrolled, and neglected zoonotic disease. Climate change is contributing to its ongoing global expansion. The dog is the main reservoir; hence the importance of implementing effective treatment, prevention, and control measures in this animal species to protect public health. However, although the standard treatment for canine leishmaniosis (CanL) is effective, it does not provide full parasitological clearance, and side effects and drug resistance have been described. The host’s immune system plays a key role in the establishment and evolution of leishmaniasis. Dietary nucleotides modulate the immune response and, given their reported efficacy and safety in sick and clinically healthy *Leishmania*-infected dogs and because they represent a sustainable option with no associated side effects or resistance, they could be included within the prevention, treatment, and control strategies for leishmaniasis. This article briefly summarizes the scientific literature on CanL management, including unresolved issues, and reviews the scientific evidence on immunomodulatory effects of dietary nucleotides in different animal species. It also proposes a CanL management algorithm, including nucleotides. It is concluded that nutritional modulation of the immune response with nucleotides can contribute to better management of leishmaniasis following a One Health approach, especially in the COVID-19 era.

## 1. Canine Leishmaniosis and Associated Immune Responses

### 1.1. Canine Leishmaniosis

Leishmaniasis is a complex of infectious diseases caused by vector-borne protozoan parasites belonging to species of the genus *Leishmania*. It is primarily zoonotic with the exception of *Leishmania donovani* and *Leishmania tropica*, and it is currently listed as an emerging, uncontrolled and severely neglected disease by the World Health Organization (WHO) [1,2,3,4]. It was first observed by William Boog Leishman in India in 1900 and later described by Leishman [5] and also by Charles Donovan [6] in 1903.

*Leishmania infantum* is mainly transmitted by the bite of parasite-infected phlebotomine sand flies causing severe diseases in different mammalian hosts, including zoonotic leishmaniasis in humans and canine leishmaniosis (CanL) in dogs [7,8,9,10,11,12,13]. Besides vectorial transmission, vertical transmission and transmission by blood transfusion have been described as well, and recently even in utero transmission were also reported [14]. The domestic dog is the primary reservoir of *L. infantum*, although other mammalian reservoirs also exist [1,15,16,17,18]. It has been suggested that cats, rabbits, and ferrets may act not only as accidental hosts, but also as reservoirs [2,19,20,21,22]. Cats are gaining importance in the zoonotic cycle of visceral leishmaniasis, as they have been shown to be capable of transmitting *L. infantum* to sand flies, and reports of feline leishmaniosis cases are increasing [23,24]. This parasite is also widespread in wild carnivores, and the risk of infection in wildlife animals is high [25,26].

Infected dogs can develop clinical disease (sick dogs), but they can also remain in the subclinical stage (clinically healthy infected dogs) [7,9,27]. Sick dogs feature clinical signs and/or clinicopathological abnormalities associated with leishmaniosis, which can affect any organ, tissue, or body fluid, with skin lesions being the most frequent manifestations [7,28,29], and which might be age-specific in some cases [30]. On the other hand, in endemic areas, there are also infected dogs, which do not feature clinical signs and/or clinicopathological abnormalities. These cases of subclinical infection are actually more frequent than those with clinical disease [31,32,33,34]. CanL asymptomatic patients are at risk of progressing into sick patients; therefore, from a veterinary and public health perspective, they should be monitored, especially in endemic areas [11,35]. The dog’s clinical condition can also influence the infection and infectivity of sand flies [36].

The diagnosis of CanL is complex, and it combines, besides compatible history and clinical signs, serological, molecular, and parasitological methods [7,29,37,38,39]. Clinical staging, either following the LeishVet guidelines for the practical management of CanL [7] or the guidelines for the treatment of leishmaniasis in dogs [40], is also a very useful tool, especially during patient monitoring [41], as clinical evolution can have an impact on the prognosis and influence the efficacy of a particular treatment protocol. In an attempt to optimize the detection of infected dogs and to improve the way CanL patients are diagnosed and monitored, several innovative techniques have been developed over the past few years, such as parasite detection and measurement of other immune system biomarkers from saliva samples [42,43,44,45]. Furthermore, it is also expected that in the future, more widespread use of serum measurement of circulating immune complexes will contribute to improving diagnosis, evaluation of disease progression, and monitoring response to treatment [46].

### 1.2. Immune Response and Disease Prognosis

The outcome of patients with leishmaniasis is influenced by several factors involving parasite features, vector biology, and host. Among these, immune responses are a key factor [1,4,47]. In humans and dogs, the protective immunity against leishmaniasis is mediated by T cells and associated with higher production of IFN-γ and TNF-α. The predominant Th1 (cellular) immune response correlates with better resistance to disease, resolution of infection, and enhanced immunity against the disease [48,49]. This effective cellular response promotes intracellular macrophage clearance of *Leishmania* parasites [31,50]. On the other hand, disease progression has been linked to higher levels of Th2 cytokines, such as IL-4 and IL-10, and predominance of humoral response [49,51]. 

In dogs, the type of immune response against the parasite also markedly determines whether a dog develops clinical disease or remains in a subclinical stage, and it also strongly affects the prognosis [3,7,9,17,52,53,54,55,56]. It also seems that some canine breeds present a higher susceptibility to CanL (Boxer, Cocker Spaniel, Rottweiler, and German Shepherd), while the Ibizan Hound is a breed with reported resistance to *Leishmania* infection [51]. Dogs with the subclinical disease tend to show a weak or absent Th2 (humoral) immune response and stronger Th1 response, low titers of anti-*Leishmania* antibodies, and a low parasite burden, whereas sick dogs feature an exacerbated Th2 immune response and an absent or weak Th1 response along with high antibody titers and systemic parasite dissemination [7,29]. Increased bone marrow IFN-γ and TNF-α gene expression has also been described in dogs with *L. infantum* infection and suggested as a potential cause of the peripheral blood hematological changes observed in these patients [57]. 

Nonetheless, the exact immune mechanisms, which confer resistance or susceptibility to *Leishmania* infection and subsequent disease are not yet completely known in dogs [9]. Moreover, in fact, the role of different types of immunity and the specific pathways leading to disease control or disease progression in humans are not totally clear either. Instead of a simple Th1/Th2 dichotomy, it might be possible that the evolution of leishmaniasis depends more on the degree of humoral immunity, with high IgG levels being associated with parasite persistence [58].

## 2. Strategies for Treatment, Prevention and Control, and Unresolved Issues

### 2.1. Recommended Treatment Options

Leishmaniasis is currently uncontrolled and categorized as an emerging neglected disease, which is why it is necessary to promote research programs aimed at improving vector control and effective diagnostic and therapeutic options for the various affected animal species [1]. To date, unfortunately, very few drugs are available for the clinical management of leishmaniasis, either in humans or in companion animals [7,21,59,60]. The WHO recommends administering anti-leishmanial drugs for humans only to human patients and not for veterinary purposes due to the potential of drug resistance development. In particular, it specifically discourages the use of amphotericin in dogs to avoid the occurrence of amphotericin B-resistant *Leishmania* strains in humans [40]. However, similar compounds are still used in human and veterinary medicine for treating humans and CanL, including mainly pentavalent antimonials, allopurinol, and miltefosine. Therefore, care should be taken to reduce the risk of developing resistance [61,62,63,64]. Immunomodulators are considered one of the keys to the future of leishmaniasis treatment, and fortunately, novel therapeutic options are under investigation [59,63,65,66,67,68].

In dogs, there are different published guidelines for the treatment of leishmaniosis, namely the guidelines for the treatment of leishmaniasis in dogs by the Canine Leishmaniasis Working Group [40] and the guidelines for the practical management of canine leishmaniosis by the LeishVet group [7]. They both agree on the first line and most effective treatment for CanL, which is currently a combination of subcutaneous N-methylglucamine antimoniate (MGA) for 4–6 weeks with oral allopurinol for at least 6 months [11,61,69,70]. This is also the treatment protocol followed by most veterinary practitioners [71,72,73]. MGA is a pentavalent antimonial with parasiticidal activity, which potentiates the phagocytic capacity of macrophages, leading to a parasite load reduction in infected dogs [69,74]. Potential side effects of MGA include nephrotoxicity and cutaneous abscesses, and cellulitis [56,70], although no remarkable negative impacts have been observed after short-term treatment [75]. Allopurinol, a purine analog of adenosine nucleotides, blocks RNA synthesis in *Leishmania* parasites, which inhibits parasite multiplication. However, allopurinol treatment also carries limitations. It has been related to some side effects, mainly xanthine urolithiasis due to increased urinary xanthine levels [11,76,77,78,79,80,81], and allopurinol resistance has been reported in dogs and associated with clinical relapse [82,83,84]. It should be mentioned, however, that to date, there is scarce scientific evidence of the effects of long-term allopurinol treatment in CanL patients.

Miltefosine is also recommended by the WHO for the treatment of post-kala-azar dermal leishmaniasis [1]. It is actually the first and only oral drug that can be used to treat leishmaniasis in people. It is a phospholipid (hexadecyl-phosphocholine), which can reduce *Leishmania* replication, but it is not able to completely remove the parasite from lymph nodes, which is why it is considered ineffective as sole treatment [69]. In dogs, miltefosine has been suggested for treating CanL patients in combination with allopurinol as an alternative to MGA plus allopurinol, but not as a sole therapy either [56,85]. In addition, it has been reported that antimonials provide a faster resolution of acute-phase proteins concentrations in CanL, compared to miltefosine [86]. As with MGA and allopurinol, side effects have also been described in CanL patients receiving miltefosine, including gastrointestinal disorders and teratogenicity [85,87,88]. In addition, in a recent study, it was concluded that treating CanL patients with miltefosine induced resistance to miltefosine and amphotericin B as well as changes in parasite fitness [89]. These effects could have a marked impact on animal and human public health. 

Lastly, in dogs, marbofloxacin has also been reported to improve the clinical score and to reduce parasite load [90].

Thus far, none of the available chemotherapies has reliably eliminated *Leishmania* infection, and standard treatment often results in clinical relapses. Unfortunately, MGA plus allopurinol or miltefosine plus allopurinol treatment combinations only temporarily improve clinical signs in dogs and do not eliminate the parasites completely [62]. In addition, their drawbacks, including high cost, undesired side effects, and development of resistances, highlight the need for their replacement by better options, as their use involves a public health risk [7,31]. Because of the scarce therapeutic arsenal available in different species affected by leishmaniasis [7,21,59], and given the potential for development of drug resistance and side effects, the search for new therapies for both humans and animals is an urgent task.

On the other hand, at present, guidelines do not recommend treating clinically healthy infected dogs because of the potential for promoting parasite resistance. In such cases, the currently suggested approach is based on monitoring their clinical status and performing periodic serological testing [7,27,32,40]. Indeed, monitoring infected patients is crucial, and response to treatment in dogs with leishmaniosis can be assessed by evaluating changes in clinical signs, and by determining serum proteins and acute-phase protein (APP) indexes [29,91], among other biomarkers [92,93]. Nonetheless, limiting treatment to sick dogs only diminishes the potential impact in reducing the prevalence of leishmaniosis in dogs and people in endemic areas because, even though some of these dogs may never develop clinical disease, they have the potential to transmit the parasite to other dogs, to human beings, and to phlebotomine sandflies [7,94,95,96,97,98]. As these dogs represent a veterinary and public health concern, their management is currently an unresolved issue, and innovative approaches to this challenge are needed [95].

CanL management could benefit from novel therapies, which, besides being safe and efficacious, would not be used in humans hence reducing the potential risk of developing drug-resistant parasites that would be passed on to people [64]. These solutions might come from alternative therapies, which are currently either under investigation or already available but not yet widely used, such as cell therapy [99], autovaccine [73], artemisinin and derivatives [66,67,100], aminosidine [101], or some types of immunotherapy [95].

### 2.2. Prevention, Control, and Public Health Considerations

Human and canine leishmaniosis is endemic in the Mediterranean Basin, the Middle East, and sub-tropical and tropical regions of the world, but over the past few years, with climatic zone shifts induced by climate change, population instability, and globalization, the disease has been experiencing a clear geographical expansion [9,102,103,104,105,106,107,108,109]. Several cases have been reported in non-endemic areas [31,110,111] such as the UK [112,113], the United States [114,115], New Caledonia [116], Germany, and Poland [110]. Besides, the incidence of CanL is increasing in endemic countries such as Spain and France [117]. Thus, prevention and control measures following a multidisciplinary and integrated One Health approach are required in order to manage this and other parasitic zoonoses [103,106,108,117,118]. In November 2020, the World Health Assembly of the WHO endorsed a road map for neglected tropical diseases 2021–2030 with the objective of preventing, controlling, eliminating, and eradicating 20 diseases and disease groups, including leishmaniasis [119]. Since dogs are the main natural reservoir of infection for humans, this species is the main target of control measures because controlling the spread of CanL should lead to a reduction in the number of cases in humans. In line with this, in a recent position paper from the World Veterinary Association (WVA) [120], two main recommendations were made regarding leishmaniasis control and prevention: (1) prevention programs should be focused on disrupting the transmission of infection and preventing canine infection from protecting animal and human health, within a One Health approach; and (2) the use of insecticides should be increased to reduce potential transmission. Although in some countries, dog culling has been used as a control measure as part of government policy, the futility of this method has been clearly stated [121], and it should, therefore, not be included within control programs. From a One Health perspective, besides dogs, other animal species, which could act as reservoirs, should also be taken into consideration [8,9,122].

Vaccination of dogs together with the use of topical insect repellents is the most effective combination to prevent and control CanL in order to, in turn, reduce the prevalence of human disease [27,51,73,98,123,124]. To date, there is no registered vaccine that prevents human leishmaniasis [1]. In dogs, however, several vaccine products for CanL have been developed [32,95,125,126,127,128]. Hopefully, the latest publications reporting their efficacy and safety, especially those performed with Letifend^®^ (Laboratorios Leti, Barcelona, Spain) [129,130], will lead to their use becoming more widespread and will ultimately contribute to improved disease prevention and control. 

Immunotherapy could play a key role in the prevention and control of leishmaniasis, and targeting the host immune response to the parasite in dogs should help improve the efficacy of vaccines and treatment protocols [95,131,132]. There are several options available, and significant advances are being made through ongoing scientific research and new developments. One of these options is domperidone, a dopamine D2 receptor antagonist, which has been shown to improve clinical signs and reduce serum antibody titers in *L. infantum*-infected dogs [133], to reduce seroconversion rates in healthy seronegative dogs by enhancing Th1 immune response [134], and to improve serum creatinine and antibody titers in a small number of dogs exposed or infected with *L. infantum* and suffering from chronic kidney disease [135]. Another option is phospholinoleate-almitoleate anhydride (P-MAPA), a product derived from *Aspergillus oryzae*, which can lead to a reduction of clinical signs and parasite load in the skin of sick dogs [136]. Domperidone and P-MAPA appear to be safe, but their efficacy remains controversial as limited data are available [95,135]. Perhaps monoclonal antibodies will provide further solutions and applications in leishmaniasis and reinforce the usefulness of following this strategy of modulating the immune response [2].

## 3. Immunonutrition and Bioactive Compounds

Immunonutrition refers to the modulation of the activities of the immune system, and the consequences for the patient of immune activation, by nutrients or specific food items fed in amounts above those encountered in the normal diet. Supplementing some specific bioactive compounds through the diet might then allow a positive modulation of the immune response [137,138]. Therefore, the use of some of these immunomodulatory nutrients could become an appropriate complementary approach in the management of several diseases with a relevant immune component, including leishmaniasis.

Nucleotides are considered immunomodulatory nutrients [137]. These low molecular weight bioactive compounds are the building blocks of DNA and RNA and are important for many physiological processes in living organisms. They can naturally be found in all foods of animal and vegetable origin as free nucleotides and nucleic acids [139,140,141]. Nucleotides are composed of a five-carbon sugar molecule, a heterocyclic nitrogenous nucleobase, either pyrimidine or purine, and a phosphate group [140]. Sources include de novo synthesis, recovery via salvage mechanisms, and dietary intake. Under normal conditions, de novo endogenous synthesis serves as the main nucleotide source in animals. However, dietary nucleotide supply becomes conditionally essential in certain situations in which nucleotide demand increases and the body is not able to produce enough to meet demand. Representative examples include physiological stress, immunosuppression, infection, and certain disease states [140,141,142]. In several animal species, exogenous nucleotide supply has been reported to lead to improved biological functions and several health benefits, including modulation of immunity, resistance to infection, promotion of growth and development, maintenance of intestinal and liver function, and promotion of cell proliferation and differentiation [141]. 

The immunomodulatory activity of dietary nucleotides could potentially be translated and applied to many benefits in animal health. In particular, several research studies provide scientific evidence supporting the positive effects of Nucleoforce^®^, a proprietary brand of nucleotide-rich yeast extract developed by Bioiberica S.A.U. (Palafolls, Spain). This nucleotide extract is a highly sustainable product as it can be obtained through a fermentation process following a circular bioeconomy approach. To date, there is limited scientific evidence reporting its effects in companion animals [143,144,145,146,147,148,149,150], but prior publications support its use in humans [151,152], aquatic species [153,154,155,156,157,158,159,160,161,162,163], and in livestock species [164,165,166,167,168,169,170,171,172,173,174,175,176,177,178] (Table 1). These studies show a beneficial impact on the immune system and disease resistance, among other beneficial effects, which could serve as background for further studying the usefulness of this product as a tool for modulating the immune response in dogs suffering from diseases such as leishmaniosis, especially given the key role of the immune response in CanL.

AHCC^®^, a standardized extract of cultured *Lentinula edodes* mycelia, is another bioactive compound with reported immunomodulatory activity. It contains polysaccharides, amino acids, lipids, and minerals, and it is especially rich in α-glucans. This product was developed by Amino Up Chemical Co. Ltd. (Sapporo, Japan) in Japan in 1992. Several articles describe its therapeutic effects in both in vitro assays and in human and animal studies, including modulation of the immune response, antioxidant and anticancer activity, and prevention of infectious processes [179,180,181,182,183,184,185,186,187]. Leishmaniasis patients could benefit from some of these reported positive effects, such as increased Th1 cell responses [179,180,181], increased production of IL-17 and IFN-γ by CD4+ T cells [180], and modulation of the immune response in intestinal epithelial cells and macrophages [183].

## 4. Nucleotides in Leishmaniasis

### 4.1. Reported Effects of Nucleotides and AHCC

Given the critical role of the type of immune response in CanL patients and their immunomodulatory benefits, and since there is an identified need for alternative and complementary solutions for the management of CanL patients, the effects of nucleotides, with or without AHCC in leishmaniasis have been evaluated over the past few years, and they are still being studied in several ongoing R&D projects. The aim of such investigations is to provide scientific evidence for the use of immunomodulatory compounds in clinical situations, which are currently unresolved. Thus far, three studies have reported the effects of nucleotides in leishmaniasis [147,148,188].

**Figure 1:**
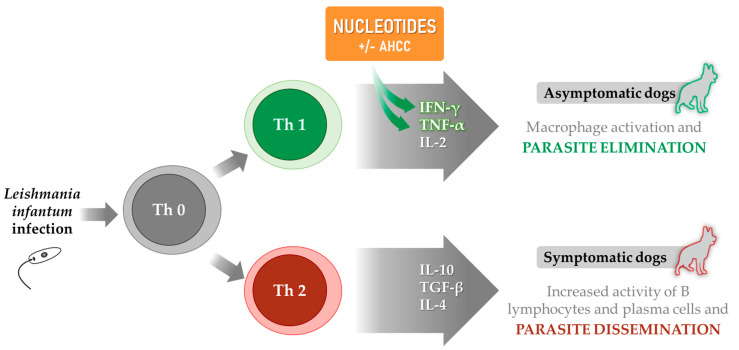
Enhancement of the effective Th1 immune response mediated by nucleotides, with or without AHCC, by increasing IFN-γ and TNF-α release. Adapted from Baneth et al. 2008 [31]; and Barbiéri et al., 2006 [17].

Several in vitro tests have been performed to explore and characterize the immunomodulatory effects and mechanisms of action of nucleotides and AHCC using naïve and *Leishmania*-stimulated murine cells [188]. In these studies, the potential leishmanicidal activity of these compounds was assessed by quantifying nitric oxide production and replication of *Leishmania* parasites. After confirming that, as expected, there was no direct effect on the parasites, the immunomodulatory activity of the nucleotides was evaluated in different cell types from mice, with or without soluble *Leishmania infantum* antigen (SLA) infection, to see whether they had an impact on cell proliferation and cytokine production. Results revealed that nucleotides, alone or in combination with AHCC, significantly increased the production of IL-1, IL-2, IL-5, IL-9, IL-10, and IL-12 in naïve immune cells, and also the release of IFN-γ and TNF-α in naïve and *L. infantum*-infected macrophage/lymphocyte cocultures (Figure 1). These findings indicate that nucleotides enhance the effective Th1 immune response against *Leishmania* [51] and, therefore, support their use as part of an immunomodulatory management strategy for patients with leishmaniasis.

The combination of dietary nucleotides with AHCC has also been tested in vivo in dogs. Taking into account the existence of two well-defined types of CanL patients for whom treatment is an unresolved issue, sick dogs and clinically healthy infected dogs, two clinical trials have been carried out to assess the potential benefits of the combination in those two specific clinical situations [147,148]. 

The first study was a multicenter open-label positively controlled clinical trial in which 69 dogs with naturally occurring CanL were randomized to receive either allopurinol (positive control group) or nucleotides plus AHCC (treatment group). Dogs in the treatment group received an oral supplement (Impromune^®^, Bioiberica S.A.U., Esplugues de Llobregat, Spain) containing a patented combination (EP2346530B1) of nucleotides (Nucleoforce^®^, Bioiberica S.A.U., Esplugues de Llobregat, Spain) plus AHCC (Immunactive^®^, Amino Up Chemical Co. Ltd., Sapporo, Japan) once daily for 180 days in addition to an initial 28-day course of injectable MGA. The supplement provided significant improvements in clinical scores and an overall amelioration in the biomarkers used to monitor response to treatment, showing similar efficacy to MGA plus allopurinol and without producing xanthinuria [147].

The second clinical trial was designed as a multicenter, randomized, double-blind, placebo-controlled trial. In this case, 46 clinically healthy dogs naturally infected with *L. infantum* were included. Results showed that 1-year administration of the same dietary supplement combining nucleotides plus AHCC allowed a significant reduction in disease progression rate compared to placebo and a decrease in the levels of anti-*Leishmania* antibodies. Disease severity was also significantly reduced in the supplement group after 180 days. Moreover, Impromune^®^ did not produce any type of crystalluria after one year, which attests to its safety and usefulness as an immunomodulatory tool in dogs experiencing problems with allopurinol-induced xanthinuria [148].

On the other hand, it should not be overlooked that leishmaniosis also affects cats [24,60,189] and that this species is now also a part of *Leishmania* life cycle in endemic areas [23]. It is, therefore, worthwhile mentioning that, besides having shown clinical efficacy in dogs, the combined use of oral nucleotides and AHCC also appears to be efficient in feline leishmaniosis. Leal et al. [149] reported the clinical case of a 12-year-old male neutered domestic shorthair cat, which improved following nucleotide plus AHCC oral administration after having developed side effects with the standard therapeutic options. The cat had been diagnosed with granulomatous rhinitis secondary to leishmaniosis, which was initially treated with allopurinol. However, the patient developed dermatological signs compatible with a cutaneous adverse allopurinol reaction. For this reason, allopurinol treatment was stopped, and MGA was prescribed. Unfortunately, although MGA treatment led to clinical improvements, the cat presented with acute kidney injury, and MGA had to be discontinued. At that point, Impromune^®^ administration was started as an alternative, given the side effects of the standard therapeutic options. This strategy eventually led to a satisfactory clinical outcome. 

Another case report also describes the use of nucleotides with AHCC in a cat with leishmaniosis as a replacement for allopurinol when the patient developed xanthinuria [150]. The combination of nucleotides and AHCC should, therefore, be considered in cats, especially given the importance of this underdiagnosed condition in this animal species [21] and also the fact that, to date, no specific treatment has been registered for feline leishmaniosis.

### 4.2. The Role of Nucleotides in CanL Multimodal Management

Managing CanL patients is complex and should also involve the recognition of the global impact that it inevitably has on human leishmaniasis and public health. A rational approach to CanL management, based on a combination of therapeutic tools, which are safe, with no side effects, and with a low potential for developing resistances, becomes especially necessary in the globalized world of today. 

The current guidelines for the management of CanL [7,40] are very useful and have been used by veterinary practitioners successfully for many years [71], but these recommendations include treatments with associated side effects, as well as some limitations in particular clinical situations, as described earlier in this article.

As suggested by some authors, the future of CanL management should combine parasiticidal and parasitostatic treatments to eliminate the parasite, together with immunomodulators aimed at achieving a more appropriate and efficient immune response against the parasite [190]. Indeed, immunotherapy should contribute to drug sparing, combating drug resistance, and reducing the side effects of several therapeutic agents [2]. Nucleotides have been included within the choices of effective immunotherapies for the management and control of CanL in recently published articles [121,191], and other examples of nutritional modulation of the immune response with promising results in leishmaniasis also exist [192,193,194,195]. 

**Figure 2:**
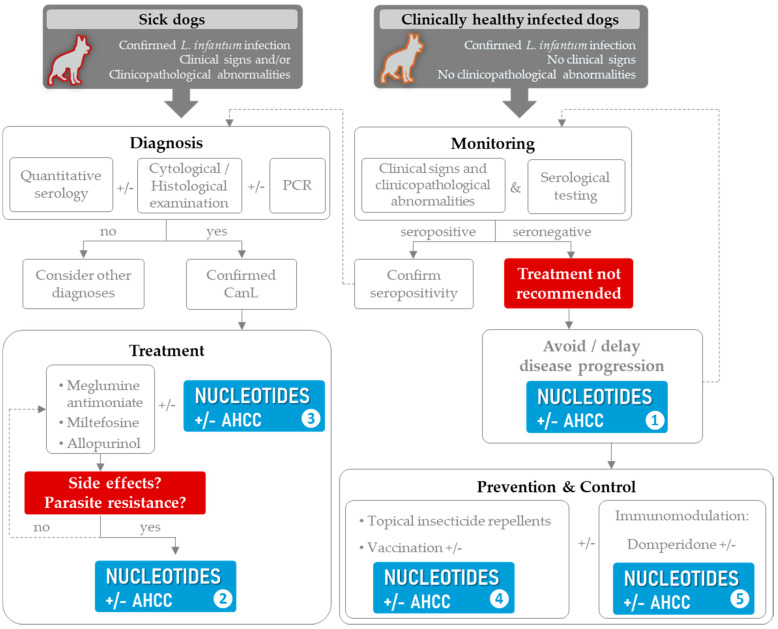
Suggested algorithm for the multimodal management strategy for CanL patients in endemic areas incorporating dietary nucleotides (with or without AHCC) as adjunctive therapy. Unresolved issues are depicted in red. Potential clinical situations in which nucleotides +/− AHCC might be useful are shown in blue; as a preventive tool to avoid disease progression in clinically health infected dogs (❶), as an alternative to allopurinol in patients with xanthinuria and/or to avoid parasite resistance (❷), as standard treatment enhancer and/or drug sparing agent (❸), as a potential enhancer of vaccine performance (❹), or as part of a prevention and control strategy, alone or in combination with domperidone (❺). Modified from Solano-Gallego et al., 2011 [7].

Based on this assumption and on the abovementioned scientific evidence and background supporting the effects of dietary nucleotides, an algorithm for the approach to managing CanL patients, including nucleotides (with or without AHCC) is proposed (Figure 2). It is suggested that nucleotides become a component of the multimodal strategy, which must necessarily be tailored to every single patient and their particular needs and clinical status. Rather than positioning nucleotides as a substitute for other already-existing therapeutic options as a rule or in any clinical situation, this protocol is aimed at providing veterinarians with a wider range of tools, which can be appropriately combined thus that treatment can be individually optimized, side effects avoided, and drug resistances minimized. 

Dietary nucleotides could be considered as an alternative to allopurinol in patients with xanthinuria. Hyperxanthinuria induced by allopurinol is a potential side effect of CanL treatment, and it involves an increased risk of developing nephrolithiasis, which may require surgical intervention. When xanthinuria occurs, possible solutions include discontinuing allopurinol administration, reducing its dosage, increasing its administration frequency, or replacing it with other therapeutic agents [196,197,198,199]. Moreover, xanthine in urine is known to enhance the in vitro multiplication of *Leishmania* [200]. Reducing allopurinol administration could help reduce the incidence of xanthinuria in these patients, although its clinical efficacy could then also be affected. Since there is evidence showing that 6-month oral nucleotides plus AHCC in addition to MGA lead to similar clinical efficacy than allopurinol plus MGA, and without involving an increased incidence of xanthinuria [147], nucleotide supplementation could be used as an alternative to allopurinol, especially in those patients showing urinary complications attributable to allopurinol treatment. In line with this, in a recent survey performed in Portugal and Spain, when veterinary practitioners were asked “If a dog under allopurinol treatment has xanthinuria, what do you do?”, most of them answered that they stop allopurinol treatment, but 34% and 16% of Spanish and Portuguese vets, respectively, said that they replace allopurinol with Impromune^®^ [199]. From a public health perspective, the use of nucleotides as an alternative to allopurinol would also pose another advantage, which is a reduction in the risk of potential development of resistance to allopurinol and the consequent associated risk of enhanced transmission of infection from dogs to humans or to other dogs [82,83,84]. Similarly, the use of dietary nucleotides as a replacement for antibiotics/trace elements has also been proposed in piglets [201].

A nutritional approach with dietary nucleotides could also be used as adjunctive treatment and as a sparing agent. In fact, a recent survey performed in Spain reports the use of Impromune^®^ by veterinarians as one of the treatment options for CanL sick patients [73]. A study with a similar product and nutritional approach has shown that an immune system-modulating diet can improve the immune response in dogs with leishmaniosis following the standard pharmacological treatment [194]. Other nutritional adjuvants have also been proven helpful in such cases by regulating inflammation and oxidative stress, as reported with polyunsaturated fatty acids and vitamins when given together with anti-*Leishmania* drugs in CanL patients [193]. Dietary nucleotides have been suggested as one of the immune therapy options to reduce the infectiousness of treated dogs as well [121]. It has also been argued that dietary nucleotides could be administered to stage I sick CanL patients together with adequate monitoring and, or instead of, allopurinol or domperidone [202].

On the other hand, given the reported 72% success rate of the LetiFend^®^ vaccine in the prevention of confirmed cases of leishmaniosis in endemic areas [129], and the known effects of dietary nucleotides, a combination of both might result in enhanced prophylactic efficacy. This possibility should be investigated, and the compatibility and potential beneficial effects of the combination of these two interventions studied.

### 4.3. Limitations and Unexplored Paths

Although the presented data and overall scientific evidence support the potential inclusion of dietary nucleotides as adjunctive treatment or as one of the many different components of the multimodal approach to the management of CanL, some limitations exist. First, although the study in sick dogs [147] provides interesting efficacy findings and a sound alternative in patients in which allopurinol administration is less appropriate, the effects after a longer follow-up period should be studied in order to characterize these potential benefits better. In such studies, adding clinical staging [7,29,40] would also contribute to the robustness of the outcomes. It would also be of interest to compare the specific effects of nucleotides with those of allopurinol thus that MGA would be out of the equation and a clear clinical benefit could be assessed and directly attributed to nucleotides. In further trials with longer-term treatment, a possible MGA-sparing effect should also be studied.

Furthermore, the study with nucleotides and AHCC in clinically healthy infected dogs [148] also has some limitations, with the small sample size being the main one. It could also be argued that, in that study, washout periods for leishmanicidal and leishmaniostatic drugs were perhaps too short. Another limitation of both clinical trials is that, thus far, all in vivo evidence in leishmaniosis refers to the combination of nucleotides with AHCC. Therefore, there is a lack of clinical studies investigating the effects of dietary nucleotides alone in CanL. Even if in vitro data point towards a positive impact of nucleotides on cell-mediated immune response by themselves [188], confirmatory in vivo studies in cases with naturally-occurring CanL is needed.

Regarding the level of understanding and characterization of the beneficial effects of nucleotides, with or without AHCC, their mechanisms of action in this particular disease, and in general, are not yet fully known even if it has been proven that they have a positive impact on humoral (serological improvements) and cellular immune responses [147,148]. Although the in vitro studies performed with combinations of such products helped to shed light on our understanding with regards to how they modulate the immune system in leishmaniosis [188], further and more thorough studies are warranted. 

The currently available scientific evidence is a good starting point, and it opens the door to many paths yet to be explored. One of these is currently going on; the GALILEI (doG triAL with Impromune in LEIshmaniosis) is a multicenter, randomized, double-blind placebo-controlled study (data on file) aimed at evaluating whether the addition of Impromune^®^ to the standard treatment protocol for CanL (MGA with allopurinol [7,40]) can improve the clinical outcomes in dogs over two years (Figure 2, clinical situation ❸). This investigation is underway, and it is expected to provide further scientific evidence on the effects of these compounds and also to cover some of the limitations of the study in sick dogs [147], which is the potential MGA-sparing effect of such a combination. Given that MGA treatment requires daily injections and has some associated side effects, if adding this nutritional immunomodulator proves to lead to a reduction in clinical relapses and a lesser need for the administration of MGA cycles, globally, the new protocol including Impromune^®^ would represent an easier treatment strategy for owners and might improve their adherence to treatment. The GALILEI study also incorporates clinical staging [11] of dogs, which is a key parameter that was not included in the previous trial in sick dogs [147]. What is more, this ongoing study also seeks to redress another limitation of the currently available published clinical trials, which is the small sample size; in this case, a special effort is being made to recruit a larger number of study subjects. Finally, in an attempt to minimize possible interferences of prior treatments in the evaluations made during the study, in the GALILEI study, washout periods for leishmanicidal/leishmaniostatic drugs or other immunomodulatory products have been extended, compared to previous trials [147,148], from two to four months. Nonetheless, given the clinical evolution of this disease in dogs, even longer (more than two years) treatment duration and more extended study follow-ups would be required to fully assess the usefulness of such intervention long term.

Based on the already-existing scientific evidence and the knowledge on nucleotides, an interesting path to be explored would be the management of healthy seronegative dogs living in high leishmaniosis-prevalence areas. As mentioned before, domperidone has shown preventive effects in this kind of patient but its administration has been associated with some side effects, such as galactorrhea and gastrointestinal disturbances [134], and care should also be taken by veterinarians given the potential cardiotoxicity of domperidone when given together with drugs that inhibit CYP450s liver enzymes or those that prolong the QT interval [203]. Moreover, given the nature and mechanism of action of domperidone, potential effects on the hypothalamus-hypophysis axis must be taken into consideration [204,205,206]. Based on the known mechanism of action of nucleotides [188], they could be useful for managing such types of patients.

Another interesting potential path to be explored is leishmaniosis in cats. Underdiagnosed but increasingly reported around the world, the disease also affects cats [24,60,132,207]. In endemic areas, there is an association between feline immunodeficiency virus (FIV) and *L. infantum* infections, and these cats are suspected of having an underlying immune system impairment due to concurrent FIV infection (alone or together with feline leukemia virus (FeLV) infection), cancer, diabetes mellitus, or caused by an autoimmune disease or treatment with immunosuppressive drugs [207]. Compared to CanL, information about feline leishmaniosis management is scarce and inconsistent. In cats, similar therapeutic agents are used, which offers up similarly associated drawbacks such as side effects and potential drug resistances [60,208]. Up to now, besides the feedback and clinical experiences of veterinary practitioners [149,150], there is no robust evidence to support the effective use of nucleotides in feline leishmaniosis. However, given the evidence-based data in vitro and in dogs, it would make sense to assess the potential benefits provided by such treatment in this species.

Yet another option worth exploring is the administration of nucleotides as a component of a petfood prescription diet especially designed for leishmaniosis. Thus far, the approach in CanL patients has followed a once-daily oral administration of a supplement containing nucleotides and AHCC, which comes in palatable tablets or as an oral paste (www.impromune.com, accessed on 8 December 2021). Incorporating nucleotides within the daily diet for dogs and cats living in endemic areas would allow easier, long-term administration of such nutritional immunomodulator compounds, improving owner treatment adherence.

A more remote but still reasonable path to be explored is the administration of dietary nucleotides to human patients with leishmaniasis, either as part of the treatment protocol or as a preventive tool. This would make sense, based on the mechanism of action of nucleotides in leishmaniasis as we understand it [147,148,188], and also given the safety and beneficial immunomodulatory effects of nucleotides observed in people [151,152].

Furthermore, and given the importance of cutaneous leishmaniasis in people and companion animals, topical application of nucleotides could also be studied in both humans and in dogs with skin lesions. Needless to say, these possibilities should be properly investigated, and the necessary studies should also be performed accordingly.

## 5. Future Perspectives

Thus, where are we heading? If we think about the future, all considerations described in this article should necessarily be taken into account within the context of the current global situation. Some of the key conditioning factors of such a situation include all the social, economic, and political elements affecting the global evolution and control programs for leishmaniasis, as well as the impact of global warming and recent disease outbreaks.

Despite the increased global awareness of leishmaniasis and the implementation of control strategies, as well as agreements on following road maps for this neglected zoonotic disease during the last few years, for humans, there are today few therapeutic options and suboptimal diagnostic tools in some areas of the world. Prevention and control remain priority needs [1,2,51,104]. Although the number of cases has been decreasing in the past few years in severely affected countries such as India, Nepal, and Bangladesh, it is still very difficult to make an estimate on a potential date for the complete and definitive elimination of leishmaniasis globally. Major obstacles to achieving this purpose are post-kala-azar dermal leishmaniasis (PKDL) and human immunodeficiency virus (HIV) coinfections [2,4,59,209]. In a globalized world, global consciousness is of paramount importance, and global and coordinated actions should be mandatory to achieve effective control of leishmaniasis.

Climate change has affected the transmission of vector-borne diseases, contributing to the ongoing expansion of leishmaniasis, and it will continue to do so. In the future, together with globalization and other factors such as vector dispersion via international air travel [107], the alteration of climatic zones driven by global warming might become even worse, and vector-borne disease outbreaks may well occur. On the bright side, even if improved and extended climatic suitability for sandflies helps extend the leishmaniasis risk to non-endemic countries, some models also predict that other regions might become too hot and humid for the vector [107]. Effective epidemiological surveillance of vector-borne zoonotic infections such as leishmaniasis needs to be taken into consideration in addition to vector control. 

Lastly, another consideration to bear in mind when facing the future is the impact of disease outbreaks, such as the recent one generated by the severe acute respiratory syndrome coronavirus 2 (SARS-CoV-2), which causes the disease now known as coronavirus disease 2019 (COVID-19) and led to a global outbreak, which has been classified as a pandemic [210]. In a recent report by the WHO, the impact of the COVID-19 pandemic on seven neglected tropical diseases is analyzed. Visceral leishmaniasis is one of the diseases for which the models used in this analysis suggest that remedial strategies are most likely to be needed due to the pandemic [211]. This crisis has reminded the world’s population of our vulnerabilities, and has severely affected socioeconomic, political, and public health domains in many countries. One of these vulnerabilities is precisely the endemicity for neglected zoonotic diseases, such as leishmaniasis. Special care should be then taken because, while the true incidence of visceral leishmaniasis is increasing, the observed incidence of the disease may decrease [212]. Indeed, the COVID-19 pandemic has put the focus on zoonotic diseases, the need for the implementation of effective measures on a One Health basis, and on our current global lack of preparedness to react when such emergencies occur. This pandemic has highlighted the importance of the link between animals and people and of working in a coordinated and translational manner [213,214,215]. Hopefully, despite the damage that it has unfortunately already caused and is still causing, COVID-19 might contribute to increasing awareness of the risks posed by zoonoses and lead to a greater commitment to One Health, potentiating closer collaborations between the veterinary and human research communities, as well as cooperation with conservation and environmental officers. With that, if in the future we need to face a new pandemic, a proactive approach might be possible thus as to provide better outcomes than the current reactive actions undertaken to fight COVID-19.

Attempting to solve all these major problems with the solutions described in the present article would clearly be beyond its scope. However, every little bit helps, and surely it would not be an overstatement to suggest that promoting innovative, sustainable, safe, and effective solutions, such as nucleotides, might contribute to enhancing the future success of all these coordinated efforts.

## 6. Conclusions

The control of *Leishmania* infection in the canine population is fundamental in order to avoid the spread to other dogs, sand flies, and humans. Prevention of CanL should help reduce the prevalence of human leishmaniasis in endemic areas. Since the current standard treatment for CanL comes with some limitations and is not always effective, novel solutions are needed.

Scientific evidence supports the use of dietary nucleotides, with or without AHCC, in CanL. This article presents a multimodal management algorithm for CanL in which the inclusion of nucleotides offers a wider therapeutic arsenal for veterinarians. The incorporation of nucleotides within the guidelines for the management of CanL could also contribute to drug sparing, combating drug resistance, and to reducing side effects associated with the standard treatment. Potential clinical situations in which dietary nucleotides might be useful include their use as a preventive tool to avoid disease progression in clinically healthy infected dogs, as an allopurinol alternative option in patients with xanthinuria and/or to avoid parasite resistance, as a standard treatment enhancer and/or drug sparing agent, as a potential enhancer of vaccine performance, and as part of the prevention and control strategy.

We need to strengthen leishmaniasis prevention and control programs, while a nutritional modulation of the immune response with nucleotides may contribute to better management of leishmaniasis based on a One Health approach, especially in the era of COVID-19.

## Figures and Tables

**Table 1:** Scientific literature reports the in vivo immunomodulatory activity and health benefits provided by the oral administration of the nucleotide-rich yeast extract Nucleoforce^®^ in several animal species.

Animal Species	Main Effects	Reference
Domestic dog, *Canis familiaris*	Increased antibody titers against parvovirus 14 days post-vaccination, higher unspecific immunoglobulin levels, and improved peripheral blood mononuclear cells test in puppies at weaning.	Romano et al., 2007 [143]
Domestic dog, *Canis familiaris*	Increased lymphocyte proliferation and higher levels of IgA, IgG, and IgM in dogs receiving chemotherapy treatment (in combination with AHCC).	Evangelio et al., 2008 [144]
Domestic dog, *Canis familiaris*	Improved leukopenia and neutropenia associated with chemotherapy, increased IgA and IgM levels, and expansion of CD3 and CD4 lymphocytes.	Burkhart et al., 2011 [145]
Domestic dog, *Canis familiaris*	Clinical and parasitological improvements in two cases of canine demodicosis unresponsive to ivermectin (in combination with AHCC).	Bernal et al., 2014 [146]
Domestic dog, *Canis familiaris*	In dogs with clinical leishmaniosis receiving an initial course of MGA, clinical superiority vs. allopurinol after 6 months, without producing xanthinuria (in combination with AHCC).	Segarra et al., 2017 [147]
style="border-bottom:solid thin">Domestic dog, *Canis familiaris*	style="border-bottom:solid thin">In clinically healthy *L. infantum*-infected dogs, significant reduction in serology and disease progression rate after 1 year (in combination with AHCC).	style="border-bottom:solid thin">Segarra et al., 2018 [148]
Domestic cat, *Felis silvestris catus*	Clinical efficacy of nucleotides and AHCC combined with miltefosine in a cat with leishmaniosis, which had developed side effects following treatment with allopurinol, as well as side effects to MGA treatment.	Leal et al., 2018 [149]
style="border-bottom:solid thin">Domestic cat, *Felis silvestris catus*	style="border-bottom:solid thin">Use of nucleotides with AHCC in a cat with leishmaniosis, which had developed xanthinuria secondary to allopurinol treatment.	style="border-bottom:solid thin">Domínguez et al., 2019 [150]
Human, *Homo sapiens*	Protective effect on biomarkers of immune response in athletes after four weeks of strenuous exercise.	Casajús et al., 2009 [152]
style="border-bottom:solid thin">Human, *Homo sapiens*	style="border-bottom:solid thin">Beneficial effect on biomarkers of immune response in athletes after four weeks of strenuous exercise under a cold environment.	style="border-bottom:solid thin">Riera et al., 2013 [151]
Nile tilapia, *Oreochromis niloticus*	Increased survival upon exposure to *Aeromonas sobria*, and improved levels of blood proteins, leukocytes, antioxidant activity, non-specific immunity, cytokines, and gene expression.	Reda et al., 2018 [153]
Nile tilapia, *Oreochromis niloticus*	Improved body protein and fat content, and increased expression of ghrelin and insulin-like growth factor genes.	Selim et al., 2020 [154]
Striped catfish, *Pangasianodon hypophthalmus*	Increased lymphocytic proliferation activity, nitric oxide concentration and lysozyme activity, and improved resistance to *Pseudomonas aeruginosa* challenge.	Yaseen et al., 2020 [156]
Gilthead seabream, *Sparus aurata*	Improved performance parameters, positive impact on liver enzymes, improvements in gene expression, and modulation of gut microbiome.	El-Nokrashy et al., 2020 [157]
Pacific white shrimp, *Litopenaeus vannamei*	Positive impact of nucleotides on the immune system and disease resistance against *Vibrio harveyi* in Pacific white shrimp.	Novriadi et al., 2021 [158]
Gilthead seabream, *Sparus aurata*	Increased gut associated lymphoid tissue (GALT) and enhanced leucocyte phagocytic capacity.	Borda et al., 2005 [159]
Gilthead seabream, *Sparus aurata*	Improved performance parameters, including final weight, feed conversion rate, and growth efficiency.	Estruch et al., 2015 [160]
European sea bass, *Dicentrarchus labrax*	Improved performance and biochemical parameters as well as improved gastrointestinal histological evaluation.	Magouz et al., 2021 [161]
Atlantic salmon, *Salmo salar*	Reduced mortality and improved immune response upon challenge with *Piscirickettsia salmonis*	Borda et al., 2008 [162]
Meagre, *Argyrosomus regius*	Increased relative growth rate in meagre fed diets with high levels of vegetable proteins.	Sáenz de Rodrigáñez et al., 2012 [163]
style="border-bottom:solid thin">Largemouth bass, *Micropterus salmoides*	style="border-bottom:solid thin">Improved histomorphology and enhanced expression of genes associated with immune response in juveniles fed with soybean-based diets.	style="border-bottom:solid thin">Romano et al., 2021 [155]
Piglets, *Sus scrofa*	Prevention of post-weaning diarrhea and attenuated reduction of villous height in weaned piglets.	Martínez-Puig et al., 2007 [164]
Piglets, *Sus scrofa*	Protective effect on intestinal cells against increased membrane permeability caused by enterotoxigenic *Escherichia coli.*	Roselli et al., 2007 [175]
Piglets, *Sus scrofa*	Modulation of gut microbiota composition in piglets after weaning, acting especially in the ileum.	Andrés-Elías et al., 2007 [176]
Piglets, *Sus scrofa*	Supplementation before weaning can improve the adaptive capabilities of weaned piglets to stressors, enhancing their growth performance.	Superchi et al., 2012 [165]
Piglets, *Sus scrofa*	Nucleotide supplementation in sows one week before farrowing until weaning significantly improves the performance of the weaned piglets.	Borda et al., 2015 [171]
Piglets, *Sus scrofa*	Nucleotide supplementation in sows one week before farrowing until weaning significantly improves the health and development of the small intestine of piglets at weaning.	Palomo et al., 2015 [172]
Piglets, *Sus scrofa*	Dietary nucleotide supplementation in sows during lactation results in the transmission of nucleotides to their piglets, leading to improvements in performance parameters and reduced mortality rates.	Segarra et al., 2017 [173]
style="border-bottom:solid thin">Piglets, *Sus scrofa*	style="border-bottom:solid thin">Nucleotide transmission from sows to piglets, allowing significantlyimproved growth and consumption by weaned piglets.	style="border-bottom:solid thin">Borda et al., 2018 [174]
style="border-bottom:solid thin">Calves, *Bos taurus*	style="border-bottom:solid thin">Reduced incidence of respiratory upset during transition from liquid to solid feeds.	style="border-bottom:solid thin">Bach et al., 2009 [178]
style="border-bottom:solid thin">Calves, *Bos taurus*	style="border-bottom:solid thin">Improved parameters related to immunity and health of the reproductive system.	style="border-bottom:solid thin">Rodríguez-Prado et al., 2017 [166]
Broiler chicken, *Gallus gallus domesticus*	Improved performance parameters during the first 21 days of life, including increased body weight and enhanced feed–to-gain ratio.	Esteve-Garcia et al., 2007 [167]
Broiler chicken, *Gallus gallus domesticus*	Increase in length of intestinal villi.	Khedr et al., 2020 [168]
Broiler chicken, *Gallus gallus domesticus*	Nucleotide supplementation counteracted the negative effects of *C. perfringens* challenge and led to the improved intestinal barrier function and intestinal histomorphology, with positive impact on performance.	Mohamed et al., 2020 [169]
style="border-bottom:solid thin">Broiler chicken, *Gallus gallus domesticus*	style="border-bottom:solid thin">Improve gut health and immunity during stressconditions.	style="border-bottom:solid thin">Kamel et al., 2021 [170]

## Data Availability

The datasets used and/or analyzed during the clinical studies in sick dogs and in clinically healthy infected dogs mentioned in this review are available from the author on reasonable request.
